# Effects and Mechanism of Surface Water Wettability and Operating Frequency on Response Linearity of Flexible IDE Capacitive Humidity Sensor

**DOI:** 10.3390/s21196633

**Published:** 2021-10-06

**Authors:** Woo Seok Yang, Seungoh Han, Gyu-Ri Lim, Hyun You Kim, Sung-Hoon Hong

**Affiliations:** 1 ICT Creative Research Laboratory, Electronics and Telecommunications Research Institute (ETRI), Daejeon 34129, Korea; gyuri1122@etri.re.kr (G.-R.L.); shong@etri.re.kr (S.-H.H.); 2 Department of Robotics, Hoseo University, Asan 31499, Korea; sohan@hoseo.edu; 3 Department of Materials Science and Engineering, Chungnam National University, Daejeon 34134, Korea; kimhy@cnu.ac.kr

**Keywords:** capacitive humidity sensor, fringing electric field, response linearity, water wettability, frequency, surface water condensation

## Abstract

Flexible capacitive humidity sensors are promising for low-cost, wearable, and radio frequency identification sensors, but their nonlinear response is an important issue for practical applications. Herein, the linearity of humidity response was controlled by surface water wettability and operating frequency of sensor, and the mechanism was explained in detail by surface water condensation. For a sensor with a Ag interdigitated electrode (IDE) on a poly(ethylene terephthalate) substrate, the capacitance showed a small linear increase with humidity up to 70% RH but a large nonlinear increase in the higher range. The response linearity was increased by a hydrophobic surface treatment of self-assembled monolayer coating while it was decreased by an ultraviolet/ozone irradiation for hydrophilicity. It was also increased by increasing the frequency in the range of 1–100 kHz, more prominently on a more hydrophilic surface. Based on experiment and simulation, the increase in sensor capacitance was greatly dependent on the geometric pattern (e.g., size, number, and contact angle) and electrical permittivity of surface water droplets. A larger and more nonlinear humidity response resulted from a larger increase in the number of droplets with a smaller contact angle on a sensor surface with higher water wettability and also from a higher permittivity of water at a lower frequency.

## 1. Introduction

Low-cost electrical humidity sensors are widely used to measure relative humidity (RH) for various applications including automotive, healthcare, industrial processes, and environmental monitoring [1,2]. They are divided into capacitive and resistive types, and the former has a higher market share compared to than the latter due to the advantages of good response linearity in a wide measurement range and low power consumption [1,2,3]. Commercial products of a capacitive humidity sensor are fabricated on ceramic substrates (e.g., glass or silicon) to have the sandwich structure with a solid lower electrode, a humidity sensing polymer, and a perforated upper electrode. Water vapor is transported from humid air to the sensing polymer through the upper electrode and then the electrical permittivity of the polymer increases in proportion to the amount of sorbed water, resulting in an increase in the capacitance of the sensor.

Recently, flexible humidity sensors are being developed due to their potentials for low-cost mass production using roll-to-roll (R2R) processes [4], flexible radio frequency identification (RFID) sensors [5,6], and wearable sensors for real-time monitoring of human respiration and plant transpiration [7,8]. They could be simply fabricated with low cost by printing an interdigitated electrode (IDE) on a polymer substrate (including paper [9]) acting as a humidity sensing material itself [5,6,9,10,11]. Sometimes, to improve sensor performance, a more humidity-sensitive thin film was coated on the IDE patterned substrate at the expense of fabrication complexity and cost [4,7,8].

For the conventional humidity sensors with the sandwich structure, good response linearity up to 90% RH could be obtained without much difficulty by using various sensing polymers such as polyimide (PI), cellulose acetate butyrate (CAB), poly(methyl methacrylate) (PMMA), etc. [12,13,14,15]. Their less hydrophilic nature is considered to be advantageous in obtaining good response linearity by suppressing the formation of water clusters that have a much higher permittivity (e.g., a value close to that of liquid water) than the individual sorbed molecules [15].

One the other hand, for most reported flexible humidity sensors, capacitance showed a small linear increase with RH and then a large nonlinear increase in a high RH range (e.g., above 70% RH) [4,7,8,9,10,11]. In most cases, the nonlinear humidity response depended strongly on the operating frequency of the sensors and decreased by increasing the frequency [7,8,10,11]. Interestingly, the response linearity could be improved by coating a hydrophobic film (e.g., Teflon) on a hydrophilic sensing material (e.g., CAB) [16]. This result was speculated to be due to the role of hydrophobic coating in reducing the condensation of water droplets with a very high permittivity on the hydrophilic sensor surface. Nonlinear humidity response is an important issue for the practical applications of flexible humidity sensors. However, no research has yet been conducted on the mechanism to explain the dependence of response linearity on the fabrication and operating conditions of flexible humidity sensors.

In this paper, the effects of surface water wettability and operating frequency on the response linearity were investigated for a flexible humidity sensor with a Ag IDE on a poly(ethylene terephthalate) (PET) substrate. The water wettability of the sensor surface was modified with a self-assembled monolayer (SAM) coating for hydrophobicity and with an ultraviolet/ozone (UVO) irradiation for hydrophilicity. The humidity response of the prepared sensors was measured from 1 to 100 kHz, which is wide enough to cover the full frequency range of excitation voltage for capacitive sensor interface circuits [7,9,11]. The effect of surface water droplets on sensor capacitance was also examined through experiment and finite element method (FEM) simulation, where their geometric pattern (e.g., size, number, and contact angle) and permittivity were systematically controlled. The dependence of response linearity on water wettability and frequency are well explained by the mechanism based on surface water condensation. When water droplets condense on a sensor surface in high RH, the resulting increase in sensor capacitance is dependent on their geometric pattern and permittivity, which are determined by water wettability and frequency.

## 2. Materials and Methods

### 2.1. Fabrication of Flexible IDE Capacitive Humidity Sensor

**Figure 1:**
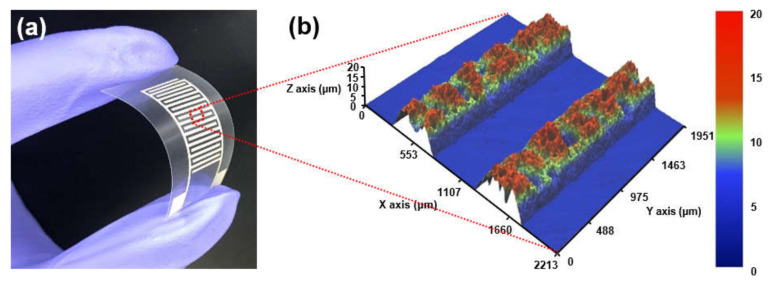
(**a**) Photograph and (**b**) 3D profile image of a flexible capacitive humidity sensor with Ag IDE on PET substrate.

A flexible capacitive humidity sensor (called as PET sensor) was fabricated on a 110-micrometer-thick PET substrate by screen printing Ag paste to form an IDE. As shown in Figure 1, the Ag IDE has 10 pairs of finger electrodes with a length of 8.5 mm, a width of 500 μm, a gap of 500 μm, and a non-uniform thickness of 11 μm on average. The PET sensor has a nominal capacitance of 2.80 ± 0.03 pF at 10 kHz in laboratory environment. To control water wettability, the sensor surface was treated using UVO irradiation and SAM coating, respectively. The UVO irradiation was carried out for 5 min in a UV/ozone cleaner (UVC-150, Omniscience) at UV wavelengths of 184.9 and 253.7 nm. The SAM was vapor-coated at 130 °C for 30 min with heptadecafluoro-1,1,2,2-tetrahydrodecyl trichlorosilane (HDFS, 95%) after the UVO pretreatment for 5 min to form a high-quality SAM [17]. Before and after the surface treatments, water contact angle (WCA) was measured at five different locations on the PET substrate and the Ag electrode using a contact angle analyzer (Phoenix 300, Surface Electro Optics, Suwon-si, Korea).

As shown in Table 1, the bare PET and Ag surfaces exhibited a low hydrophilicity with WCA of 67 ± 3° and a low hydrophobicity with WCA of 98 ± 6°, respectively. The hydrophilic PET surface was modified to have a high hydrophilicity with a WCA of 22 ± 2° by the UVO irradiation and a high hydrophobicity with a WCA of 129 ± 4° by the SAM coating. The WCA value of Ag surface was decreased to 66 ± 11° by the UVO irradiation for hydrophilicity but increased to 122 ± 3° by the hydrophobic SAM coating. It has been reported that UVO treatment forms an oxide layer to induce hydrophilicity on a polymer surface [18], and an HDFS-SAM coating covers various surfaces of metal [19], ceramic [20], and polymer [21] with CF_2_ and CF_3_ groups of HDFS to provide hydrophobicity. To be noteworthy, surface water wettability was changed more significantly on PET substrate rather than on Ag electrode by the modification treatments.

### 2.2. Measurement of Humidity Response and Analysis of Response Linearity

The humidity response of the prepared sensors was measured from 30 to 90% RH at a fixed temperature of 40 °C in an environmental chamber (TH3-KE-025, Jeiotech, Daejeon, Korea). The capacitance of sensors was measured with a precision LCR meter (E4980A, Keysight Technology, Santa Rosa, CA, USA) by applying an AC voltage of 1 V at different frequencies of 1, 10, and 100 kHz. In the operation of LCR meter, the complex impedance of sensors was measured by a 4-wire connection with coaxial cables, and the capacitance was deduced from the impedance value by using a parallel resistor–capacitor circuit model. The humidity response of capacitance was measured by increasing and decreasing RH, respectively, and then averaged for graph plotting. The humidity was changed at a slow rate of 0.33% RH/min and held for 30 min at a target value for sufficient saturation of the capacitance.

**Figure 2:**
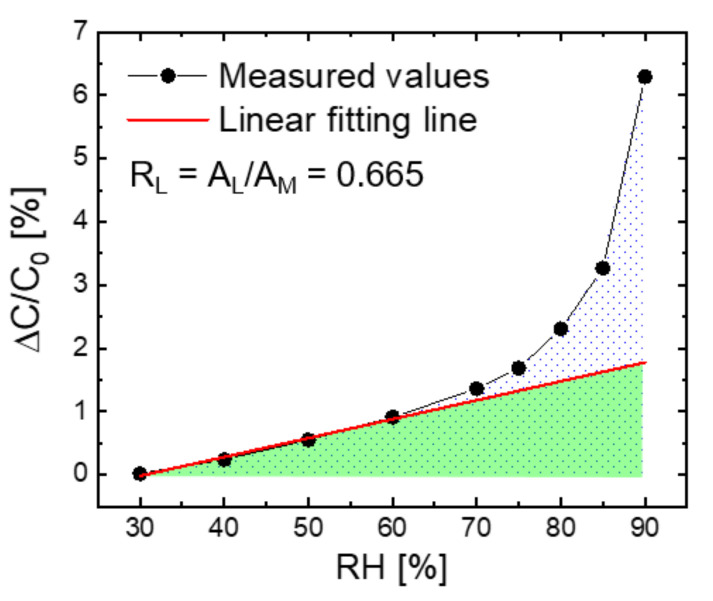
An example graph of humidity response plotted as relative capacitance change (ΔC/C_0_) with RH. The ratio of response linearity (R_L_) is defined as the ratio of the integral area of the linear fitting line (A_L_, the green-colored area) to that of the measured values (A_M_, the dotted area).

As shown in Figure 2, the measurement result of humidity response was plotted as a graph of relative capacitance change (ΔC/C_0_) with RH, and the ΔC/C_0_ value is defined as the ratio of capacitance change (ΔC) to the reference capacitance (C_0_) at 30% RH. The linearity of humidity response was quantitatively evaluated by an index of linearity ratio (R_L_), which is defined as the ratio of the integral area of the linear fitting line (A_L_) to that of the measured values (A_M_). The linear line was obtained by fitting the measured values in the range of 30–60% RH with a high coefficient of determination (e.g., R^2^ over 0.96). The R_L_ value is 1 for perfect linearity and 0 for perfect nonlinearity.

### 2.3. FEM Simulation of IDE Capacitive Humidity Sensor

During the operation of PET sensor, a fringing electric field (FEF) is formed between the finger electrodes excited with an AC voltage and penetrates to a certain extent into the air both above and below the substrate [22,23]. Thus, the sensor can be electrically described as the sensor capacitor consisting of an upper air capacitor, substrate capacitor, and lower air capacitor connected in parallel with each other. The FEF and capacitance of PET sensor were analyzed by FEM simulation using COMSOL Multiphysics software (version 5.3a). The relative permittivity (ε_r_) was set as 1 for air and 3.6 for PET. The value of ε_r,PET_ was chosen as the highest one among the reported values of 3.5–3.6 at 25 °C [24] and 3.1–3.4 at 0–80% RH and 10 °C [25] in the frequency range of 1–100 kHz.

**Figure 3:**
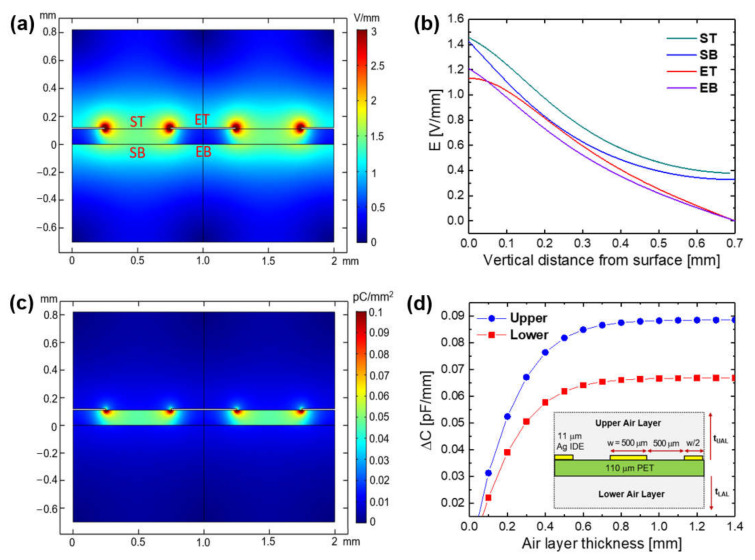
For the unit cell of capacitive sensor with 1 pair of IDEs on PET substrate in air: (**a**) the distribution of fringing electric field (FEF), (**b**) the FEF strength with the vertical distance from the sensor surfaces in different regions (ST, SB, ET, and EB), (**c**) the distribution of electric displacement field, and (**d**) the capacitance of air capacitor with the air layer thickness (blue: upper air capacitor, red: lower air capacitor).

The non-uniform FEF was formed between the IDE electrodes on PET substrate (Figure 3a), and its strength near surface decreased in the following order of region: the substrate top surface (ST) > the substrate bottom surface between IDEs (SB) >> the electrode top surface (ET) > the substrate bottom surface under IDE (EB). In the air surrounding the PET sensor, the FEF strength (E) decreased with the vertical distance from the sensor surfaces in all regions (Figure 3b) where E values were calculated at the center point of each region. At a certain distance above 600 μm, it was saturated to a value higher than 0.3 V/mm in the regions between IDEs (ST and SB), while it was continuously decreased up to almost zero in the near-electrode regions (ET and EB). Even in the regions with the same FEF strength, the electric displacement field was much higher inside the PET substrate than in the air (Figure 3c) because it is determined by the multiplication of the FEF strength and the permittivity of dielectric medium in the FEF. The capacitance of the sensor capacitor could be calculated from the FEF strength and the electric displacement field. The capacitance of the upper and lower air capacitors increased logarithmically by increasing the air layer thickness (t_UAL_ and t_LAL_) and then saturated with no further increase above a certain thickness (Figure 3d). The penetration depth [22] of the FEF between IDEs into the air above the substrate was 650 μm, where the increase in capacitance with thickness reaches 97% of the saturated value. The FEF penetration depth into the air below the substrate was 630 μm, which is 20 μm smaller than that into the upper air layer because of the substrate effect. The total capacitance of the unit sensor capacitor with 1 pair of IDE finger electrodes was 0.253 pF (100%), which was the sum of 0.089 pF (35%) of the upper air capacitor, 0.067 pF (26%) of the lower air capacitor, and 0.097 pF (39%) of the substrate capacitor.

## 3. Results and Discussion

### 3.1. Effect of Surface Water Wettability and Operating Frequency on Response Linearity

**Figure 4:**
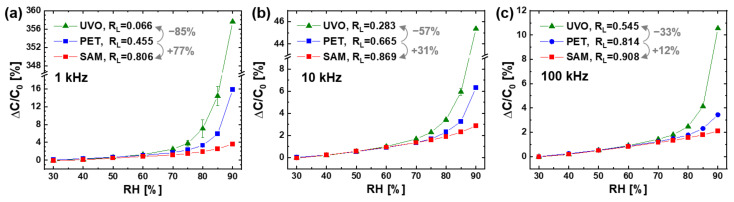
Humidity response of the PET sensors before (denoted by PET) and after the surface treatments of the UVO irradiation (UVO) and the SAM coating (SAM) at different operating frequencies: (**a**) 1 kHz, (**b**) 10 kHz, and (**c**) 100 kHz. The error bars represent the difference in capacitance values measured with an increasing and decreasing RH.

The surface treatments of the PET sensor were found to have a great effect on the linearity of the humidity response, as shown in Figure 4. At all the operating frequencies, the linearity ratio of R_L_ increased in the following order of sensor: UVO irradiated (denoted by UVO) << bare (PET) < SAM coated (SAM). As shown earlier in Table 1, the surface water wettability (inversely proportional to WCA) of the prepared sensors decreased in the following order: UVO (with WCA of 22° and 66° on PET and Ag surfaces, respectively) > PET (67° and 98°) > SAM (129° and 122°). These results show that the response linearity of the PET sensor was increased by reducing the water wettability of the sensor surface. It was reported for the capacitive humidity sensor with an indium tin oxide (ITO) IDE on a glass substrate that the response linearity could be improved by coating the hydrophobic film of Teflon (with WCA > 120°) on the hydrophilic sensing polymer of CAB [16]. Both works demonstrate the negative effect of surface water wettability on the response linearity of the IDE capacitive humidity sensor.

Our research further demonstrates that the effect of surface water wettability on the linearity of humidity response became greater at a higher operating frequency of the sensor. The increase in R_L_ by the SAM coating was 77% at 1 kHz, 31% at 10 kHz, and 12% at 100 kHz. The decrease in linearity ratio (R_L_) by the UVO irradiation was 85% at 1 kHz, 57% at 10 kHz, and 33% at 100 kHz. By the same difference in water wettability between the UVO irradiated and the SAM coated surfaces, the change in R_L_ was 12.2 times (from 0.066 to 0.806) at 1 kHz, 3.1 times (from 0.283 to 0.869) at 10 kHz, and 1.7 times (from 0.545 to 0.908) at 100 kHz.

**Figure 5:**
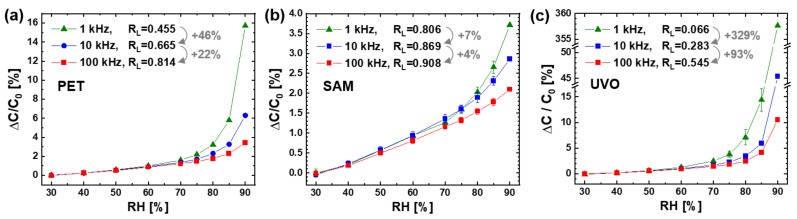
Humidity response of the PET sensors at different operating frequencies (1, 10, 100 kHz): (**a**) before surface treatment, (**b**) after the SAM coating, and (**c**) after the UVO irradiation.

For all the prepared sensors, the linearity of the humidity response always increased with the operating frequency in the range of 1–100 kHz, as shown in Figure 5. For the bare sensor with the hydrophilic PET surface (Figure 5a), the linearity ratio of R_L_ increased by 46% from 0.455 at 1 kHz to 0.665 at 10 kHz and by 22% from 0.665 at 10 kHz to 0.814 at 100 kHz. For the SAM coated sensor with the hydrophobic PET surface (Figure 5b), the R_L_ value increased by 8% from 0.806 at 1 kHz to 0.869 at 10 kHz and by 4% from 0.869 at 10 kHz to 0.908 at 100 kHz. For the UVO irradiated sensor with a higher hydrophilic PET surface (Figure 5c), R_L_ increased by 329% from 0.066 at 1 kHz to 0.283 at 10 kHz and by 93% from 0.283 at 10 kHz to 0.545 at 100 kHz.

The change of response linearity with the frequency was investigated in many papers on IDE capacitive humidity sensors [5,6,7,8,10,11]. For two similar sensors with a Ag IDE on a PI substrate, one exhibited an improvement of the response linearity by increasing the frequency from 1 to 10 and 100 kHz [10], but the other always showed a good response linearity in the high frequency range of 0.1–10 MHz [6]. For another sensor with a PI sensing polymer on a Au IDE patterned glass substrate, the capacitance at 85% RH decreased exponentially with the frequency in the low range of 1–100 kHz and then saturated with no further decrease in the high range of 100–1000 kHz [11]. On the other hand, the capacitances at 39 and 5% RH were always constant in the whole frequency range. These previous results are consistent with ours (Figure 5) in that the response linearity of the IDE capacitive humidity sensor was improved by increasing the operating frequency in the range of about 1–100 kHz.

Particularly noteworthy in our result (Figure 5) is the greater effect of frequency on the response linearity on more the hydrophilic sensor surface with a lower WCA. With the same frequency increase from 1 to 100 kHz, the improvement in R_L_ was 1.1 times (from 0.806 to 0.908) on the SAM coated surface, 1.8 times (from 0.455 to 0.814) on the bare PET surface, and 8.3 times (from 0.066 to 0.545) on the UVO irradiated surface.

### 3.2. Effects of Surface Water Droplets on Sensor Capacitance

**Figure 6:**
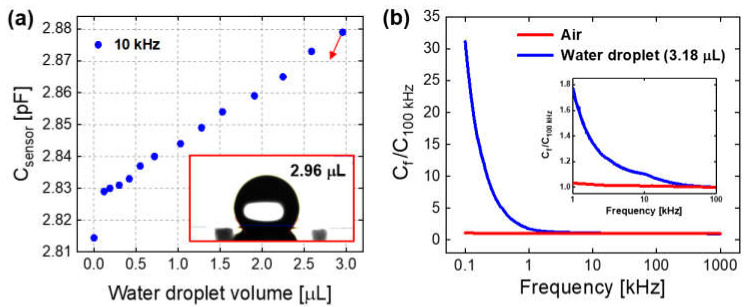
For the SAM-coated sensor with or without a water microdroplet on the top surface: (**a**) the sensor capacitance at 10 kHz with the water droplet volume (inset: optical microscope image of a 2.96-microliter droplet on the top surface of IDE patterned substrate) and (**b**) the capacitance ratio (sensor capacitance at a frequency to that at 100 kHz) with the operating frequency in the range of 0.1–1000 kHz (inset: the enlarged graph in the range of 1–100 kHz).

When a water microdroplet (2.96 μL) was formed on the top surface of the IDE humidity sensor in a laboratory environment by micropipetting, the sensor capacitance (C_sensor_) at 10 kHz was increased to 2.88 pF, as shown in Figure 6a. For the accurate measurement of C_sensor_ by using the parallel resistor–capacitor circuit model, the hydrophobic SAM coated sensor was used instead of the PET sensor where the resistance was largely reduced from 10^8^ to 10^4^ Ω by a surface water droplet. As the microdroplet evaporated, C_sensor_ decreased linearly in proportion to its volume, resulting in 2.83 pF at the smallest observed one of about 0.1μL. Finally, when the droplet had completely evaporated, C_sensor_ decreased abruptly to 2.81 pF. This result indicates that C_sensor_ increased by 0.07 pF (i.e., 2% of the initial value of 2.81 pF) because of the surface formation of a 2.96-microliter water droplet. The increase in C_sensor_ by a water droplet depended on the operating frequency, as shown in Figure 6b. The capacitance of the sensor with a water droplet on the top surface decreased exponentially by increasing the frequency. The ratio of C_sensor_ at a frequency to that at 100 kHz (C_f_/C_100 kHz_) was 31 at 0.1 kHz, 1.8 at 1 kHz, 1.1 at 10 kHz, and 0.97 at 1000 kHz. On the other hand, the capacitance of the sensor without a surface water droplet exhibited a negligible decrease with the frequency in the whole range of 0.1–1000 kHz, resulting in a C_f_/C_100 kHz_ value of 1.1 at 0.1 kHz and 0.99 at 1000 kHz.

**Figure 7:**
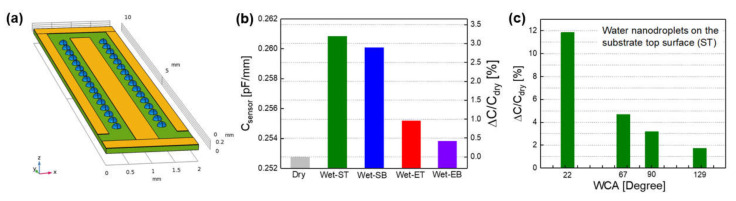
FEM simulation results on the increase in sensor capacitance (C_sensor_) according to location and contact angle of 32 water nanodroplets: (**a**) a schematic of unit capacitive sensor with the nanodroplets (blue color) between IDE finger electrodes (yellow color) on PET substrate (green color), (**b**) the increase in C_sensor_ according to the formation and location of the nanodroplets with the WCA of 90°, and (**c**) the increase in C_sensor_ with the change in WCA of the nanodroplets on the substrate top surface.

The effects of the location and contact angle of water droplets on the increase in C_sensor_ were investigated using a FEM simulation. First, 32 hemispherical nanodroplets (2.09 nL in unit volume) with a diameter of 200 μm were formed apart from IDE finger electrodes on the top surface of a PET substrate (ST region in Figure 3a) as shown in Figure 7a. For the unit sensor with one pair of IDEs, the value of C_sensor_ was 0.253 pF (C_dry_) in dry conditions without a water droplet but was increased by 3.2% (in ΔC/C_dry_) to 0.261 pF (C_wet-ST_) by the nanodroplet array (Figure 7b). When the nanodroplet array was formed on the substrate bottom surface between the IDEs (SB region in Figure 3a), ΔC/C_dry_ was relatively small at 2.9%. It was greatly reduced to 0.96 and 0.42% as the location of the nanodroplet array changed to the electrode top surface and the substrate bottom surface under IDE (ET and EB regions in Figure 3a), respectively. The contact angle of the water droplet had a greater effect on ΔC/C_dry_ than the location (Figure 7c). For the case of the nanodroplet array on the substrate top surface (ST region), ΔC/C_dry_ decreased as the WCA of droplet increased: 11.9% at 22°, 4.7% at 67°, 3.2% at 90°, and 1.7% at 129°.

**Figure 8:**
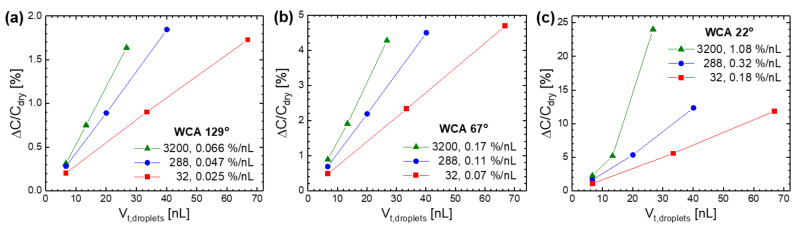
FEM simulation results on the increase in sensor capacitance according to the total volume of water droplets with different numbers (32, 288, 3200) on the substrate top surface of the IDE sensors with different WCA: (**a**) 129°, (**b**) 67°, and (**c**) 22°.

In addition to the location and contact angle, the volume number density of water droplets was found to affect ΔC/C_dry_, as shown in Figure 8. The droplets with the different WCAs (22°, 67°, 129°) were uniformly distributed on the substrate top surface (ST region in Figure 3a), and their number and size (or unit volume) changed. In most cases, ΔC/C_dry_ increased linearly with the total volume of droplets (V_t,droplets_), which is calculated by multiplying their number by the unit volume. However, it increased nonlinearly in the case of 3200 droplets with the WCA of 22°, where the droplets grew in an island manner and then coalesced to form a continuous film with increasing V_t,droplets_. For all the WCA values, the rate of increase in ΔC/C_dry_ per V_t,droplets_ increased as the number of droplets increased from 32 to 288 and 3200. This indicates that many small droplets are more effective in increasing ΔC/C_dry_ than a few large droplets.

The increase in C_sensor_ by the formation of water droplets on the IDE sensor surface (Figure 6) is because the low dielectric medium of air (ε_r,air_ = 1) in the air capacitor is partly replaced by the high dielectric water (with ε_r,water_ = 50 at 10 kHz and 26 °C [26]). As reported in prior works, ε_r,water_ decreased exponentially with the frequency in the measured range of 0.1–10 kHz [26], while ε_r,PET_ decreased slightly with the frequency in 0.1–100 kHz [24,25]. Based on these and our results (Figure 6), it is certain that water droplets on the IDE sensor surface cause the abrupt increase in C_sensor_ and the increase degree is in proportional to the amount of water droplets but in inversely proportional to the operating frequency of the sensor in the range of 0.1–100 kHz.

The dependence of the C_sensor_ increase on the location and contact angle, and the volume number density of the surface (Figure 7 and Figure 8) is due to the reason that a higher FEF strength in the region occupied by water droplets produces a larger ΔC/C_dry_. As shown earlier in Figure 3a, the FEF strength near the sensor surface decreased in the following order of region: the substrate top surface (ST) > the substrate bottom surface between the IDEs (SB) >> the electrode top surface (ET) > the substrate bottom surface under the IDE (EB). Thus, the ΔC/C_dry_ by 32 hemispherical nanodroplets was also decreased in the same order of their location: ST > SB >> ET > EB (Figure 7b). In the ST region, the FEF strength decreased by increasing the vertical distance from the surface: 1.44 V/mm at 10.9 μm, 1.41 V/mm at 25.6 μm, and 1.37 V/mm at 45.3 μm (Figure 3b). This leads to the decrease in ΔC/C_dry_ by increasing the WCA of the nanodroplets (2.09 nL in unit volume) from 22° to 67° and 129°, that is, by increasing their volume center height from 10.9 to 25.6 and 45.3 μm (Figure 7c). For the droplets with the same WCA and V_t,droplets_, their volume center height increases as their number decreases, namely, as their unit volume increases; resulting in the decrease in ΔC/C_dry_ by decreasing their number (Figure 8).

### 3.3. Mechanism for the Effects of Surface Water Wettability and Operating Frequency

The capacitance of the PET sensor (at 10 kHz) exhibited a small linear increase with RH up to 70% RH but a large nonlinear increase in the higher range (Figure 5a). The rate of increase in ΔC/C_0_ with RH was low at 0.03% per % RH in the low range of 30–70% RH but quadrupled to 0.12% per % RH in 75–80% RH and increased by 20 times to 0.6% per % RH in the high range of 85–90% RH. If surface water condensation does not occur, the sensor capacitance (C_sensor_) is calculated as the sum of capacitances of the substrate and air capacitors connected in parallel: C_substrate_ and C_air_ account for 39 and 61% of C_sensor_, respectively (Figure 3d). According to the former reports, the increase in electrical permittivity (at 10 kHz) with RH was small at 0.08% per % RH in 0–80% RH for PET [25] and was negligible at 0.0002% per % RH in 0–100% RH for air [27,28]. Based on these data, the rate of increase in C_sensor_ with RH is calculated to be 0.03% per % RH (0.08% × 0.39 + 0.0002% × 0.61 = 0.03%), which is equal to that of the PET sensor in 30–70% RH (Figure 5a). However, the high values up to 0.6% per % RH in the high range above 70% RH can never be explained by that of the normal sensor capacitor without surface water droplets.

It is well known that if the energetically favorable nucleation sites (e.g., nanocavities) are formed on a substrate surface, water condensation can occur below the saturated water vapor pressure (i.e., below 100% RH) [29,30,31]. The water condensation process is carried out in the following three stages: nucleation, growth, and coalescence. The heterogeneous nucleation was preferred on the hydrophilic region of a patterned hydrophilic–hydrophobic substrate [31,32,33]. It was predicted by the molecular dynamics simulation of water condensation that the rate of heterogeneous nucleation was higher on a surface with higher water wettability [34].

If water droplets condense on the surface of the IDE capacitive sensor at high RH, above 70% RH, the high dielectric water replaces a certain amount of the low dielectric air within the penetration depth of FEF, leading to an increase in C_sensor_ (ΔC/C_dry_ or ΔC/C_0_). Of course, a larger ΔC/C_dry_ is produced by a larger amount of condensed water at higher RH. Interestingly, at a given amount of condensed water, more droplets with a lower WCA produce a higher ΔC/C_dry_ (Figure 8). Hence, it can be explained that the condensed droplets have a larger number and a lower WCA on the surface with higher water wettability, thus ΔC/C_0_ at high RH (above the water condensation point) is higher in the following order of sensor surface: UVO irradiated (WCA of 22° on PET substrate) > bare (67°) > SAM coated (129°), similar to that in Figure 4.

**Figure 9:**
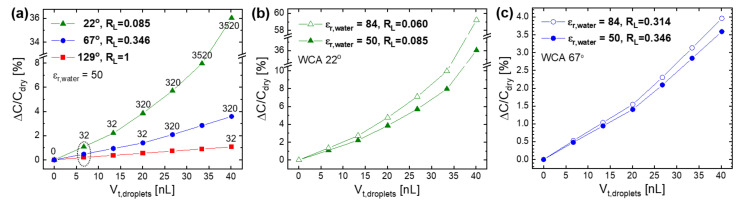
FEM simulation results showing the dependence of the increase in sensor capacitance with the total volume of water droplets and its resulting linearity ratio (R′_L_) on: (**a**) the WCA of substrate surface (22°, 67°, 129°) and (**b**,**c**) the relative permittivity of water (50, 84). It was assumed that the increase in the number of water droplets (32, 288, 3200) when increasing the total volume depended on the WCA of IDE sensor surface.

When RH continues to increase beyond the condensation point, the water droplets will increase in total volume (V_t,droplets_) through nucleation (i.e., an increase in number) and growth (i.e., an increase in size). If they only grow in size (or unit volume) without nucleation, ΔC/C_0_ will increase linearly just as ΔC/C_dry_ does with V_t,droplets_ in Figure 8. However, if V_t,droplets_ increases with RH through an increase in number as well as in size, ΔC/C_0_ can increase nonlinearly with RH similar to those (for the WCA of 22° and 67°) in Figure 9a. On the substrate top surface with the low WCA of 22°, the number of water droplets increased greatly from 32 to 3520 by increasing V_t,droplets_ from 6.688 to 40.128 nL. Here, it was assumed that V_t,droplets_ increased through the first nucleation and growth of 32 droplets, the second of 288 new ones, and the last of 3200 other ones. On the other hand, the number of water droplets increased less from 32 to 320 on the surface with the medium WCA of 67° and remained at 32 without any increase on the surface with the high WCA of 129°. This setting of simulation is plausible considering the higher rate of droplet nucleation on the surface with higher water wettability [34].

The linearity of ΔC/C_dry_ with V_t,droplets_ is quantified by another index of linearity ratio (R′_L_), that is defined as the ratio of the integral area of linear data (for the WCA of 129°) to that of nonlinear data (for the WCA of 22° and 67°). The value of R′_L_ decreased from 1 to 0.346 and 0.085 when the WCA decreased from 129° to 67° and 22°, respectively. Based on the previous result [26] and ours (Figure 6b), ε_r,water_ increases exponentially with a decreasing frequency in 0.1–100 kHz and can be roughly set as 50 at 10 kHz and 84 at 1 kHz. When ε_r,water_ increased from 50 to 84 (i.e., the frequency decreased from 10 to 1 kHz), R′_L_ decreased by 29% (from 0.085 to 0.060) on the substrate top surface with the WCA of 22° (Figure 9b), while by 9% (from 0.346 to 0.314) on that with the higher WCA of 67° (Figure 9c). The decrease in R′_L_ by the same increase in ε_r,water_ was greater on a surface with a lower WCA. This is consistent with the results of Figure 5, where the increase in response linearity with an increasing operating frequency became large on a more hydrophilic sensor surface with higher water wettability.

**Figure 10:**
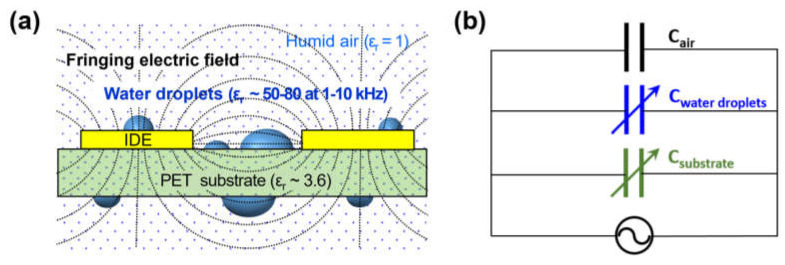
(**a**) The schematic illustration and (**b**) the equivalent circuit model of the mechanism for the effects of surface water wettability and operating frequency on the response linearity of IDE capacitive humidity sensor with PET substrate (the relative permittivities of materials are the estimated values at 1–10 kHz).

Figure 10 shows a conceptual model of the mechanism for the effects of surface water wettability and operating frequency on the linearity of humidity response of the IDE capacitive sensor with the PET substrate. During the sensor operation, FEF is formed between the IDEs to form the sensor capacitor consisting of substrate and air capacitors connected in parallel. When RH increases in a low range, the amount of water molecules sorbed from the air into the sensing material increases, resulting in small linear increases in ε_r,PET_ and, subsequently, C_substrate_. The air capacitor is involved in determining the value of C_sensor_ but rarely responds to RH. When the RH rises over a certain high value (e.g., 70% RH), water droplets condense on the sensor surface to produce C_water droplets_. As the RH continues to increase beyond the water condensation point, the droplets increase in total volume (V_t,droplets_) through nucleation and growth, namely, an increase in number and size. In this situation, more droplets can nucleate at higher RH, where more water molecules are available on sensor surface for nucleation, to produce a larger C_water droplets_ per V_t,droplets_. Hence, C_water droplets_ starts to increase nonlinearly with increasing RH. The humidity response of C_sensor_ is determined by that of C_substrate_ in the low RH range, while dominated by that of C_water droplets_ in the high RH range above the water condensation point. Therefore, ΔC/C_0_ shows a small linear increase with RH and then a large nonlinear increase in the high RH range (Figure 4 and Figure 5).

On the sensor surface with higher water wettability, the condensed droplets have a higher number and a lower WCA to produce a larger ΔC/C_dry_ (or ΔC/C_0_) at the same high RH. Additionally, their volume increase with RH becomes more dominated by nucleation (i.e., an increase in number) rather than growth (i.e., an increase in size), resulting in a larger nonlinear increase in ΔC/C_dry_ with RH. In addition, the impact of condensed water droplets on the value and linearity of ΔC/C_dry_ decreases with increasing operating frequency in the range of 0.1–100 kHz, that is, with decreasing ε_r,water_ exponentially. This frequency effect becomes more prominent at higher RH (above the condensation point) and on a more hydrophilic surface (with higher water wettability) because the condensed droplets have a larger amount and a more effective pattern (i.e., larger number and lower WCA) under such conditions.

From an electrical circuit perspective, the humidity response of the IDE capacitive sensor with the PET substrate is understood by that of the sensor capacitor consisting of the following three component capacitors connected in parallel: air, substrate, and water droplet capacitors (Figure 10b). The former is almost humidity-invariant, while the latter two are humidity-responsive. C_substrate_ increases slightly and linearly with RH over the entire range, while C_water droplets_ is formed to increase largely and nonlinearly with RH only in the high range above the condensation point. The overall humidity response of C_sensor_ is determined by that of C_substrate_ in the low RH range but dominated by that of C_water droplets_ in the high RH range. Hence, it shows a small linear increase with RH and then a large nonlinear increase in the high RH range. The value of C_water droplets_ and its nonlinear increase with RH is determined by the total volume and pattern (i.e., number and WCA) of condensed droplets as well as by ε_r,water_. Therefore, C_sensor_ (dominated by C_water droplets_) is larger and more nonlinear at higher RH, on a sensor surface with higher water wettability, and at lower frequency in the range below about 100 kHz.

## 4. Conclusions

For a flexible capacitive humidity sensor with a Ag IDE on a PET substrate, the effects of surface water wettability and operating frequency on the linearity of humidity response were investigated and their mechanism was proposed in terms of surface water condensation. The capacitance of the sensor (C_sensor_) showed a small linear increase with RH in a low range (e.g., up to 70% RH) but a large nonlinear increase in a high range. This can be explained by the fact that C_sensor_ is determined by the capacitance of the substrate (C_substrate_) in the low RH range but dominated by the capacitance of the water droplets (C_water droplets_) in the high range above the condensation point. Unlike C_substrate_, C_water droplets_ is much larger due to the high electrical permittivity of water and increases nonlinearly with humidity due to the volume increase in condensed droplets through nucleation (i.e., an increase in number) rather than growth (i.e., an increase in size). The linearity of the humidity response was increased by a hydrophobic surface treatment of SAM coating, while it was decreased by an UVO irradiation for hydrophilicity. The condensed droplets have a smaller contact angle and increase in total volume with RH through a larger increase in number on sensor surface with higher water wettability, resulting in a larger and more nonlinear increase in C_water droplets_ with RH. The response linearity was also improved by increasing the operating frequency of the sensor in the range of 1–100 kHz, because of an exponential decrease in the permittivity of water droplets with frequency in the range. The effects of surface water wettability and operating frequency were strongly interrelated: the greater the effect of water wettability at a higher frequency and the greater the effect of frequency on the sensor surface with the higher water wettability. Both effects are caused by the surface condensation of water droplets, whose geometric pattern and permittivity are determined by the water wettability and the frequency, respectively. This work will be helpful to understand and solve the nonlinear response of flexible IDE capacitive humidity sensors for their practical applications.

## Figures and Tables

**Table 1:** The WCA values on PET substrate and Ag electrode before and after the surface treatments of UVO irradiation and SAM coating.

Surface	Before Surface Treatment	After UVO Irradiation	After SAM Coating
PET	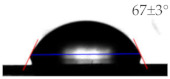	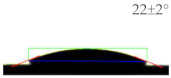	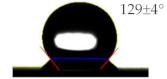
Ag	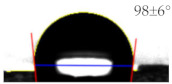	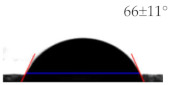	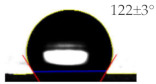

## Data Availability

Not applicable.

## References

[1] Fenner R., Zdankiewicz E. (2001). Micromachined water vapor sensors: A review of sensing technologies. IEEE Sens. J..

[2] Farahani H., Wagiran R., Hamidon M.N. (2014). Humidity sensors principle, mechanism, and fabrication technologies: A comprehensive review. Sensors.

[3] Najeeb M.A., Ahmad Z., Shakoor R.A. (2018). Organic thin-film capacitive and resistive humidity sensors: A focus review. Adv. Mater. Interfaces.

[4] Reddy A.S.G., Narakathu B.B., Atashbar M.Z., Rebros M., Hrehorova E., Bazuin B.J., Joyce M.K., Fleming P.D., Pekarovicova A. (2011). Printed capacitive based humidity sensors on flexible substrates. Sens. Lett..

[5] Oprea A., Bârsan N., Weimar U., Bauersfeld M.-L., Ebling D., Wöllenstein J. (2008). Capacitive humidity sensors on flexible RFID labels. Sens. Actuators B Chem..

[6] Rivadeneyra A., Fernández-Salmerón J., Agudo M., López-Villanueva J.A., Capitan-Vallvey L.F., Palma A.J. (2014). Design and characterization of a low thermal drift capacitive humidity sensor by inkjet-printing. Sens. Actuators B Chem..

[7] Li B., Tian Q., Su H., Wang X., Wang T., Zhang D. (2019). High sensitivity portable capacitive humidity sensor based on In_2_O_3_ nanocubes-decorated GO nanosheets and its wearable application in respiration detection. Sens. Actuators B Chem..

[8] Lan L., Le X., Dong H., Xie J., Ying Y., Ping J. (2020). One-step and large-scale fabrication of flexible and wearable humidity sensor based on laser-induced graphene for real-time tracking of plant transpiration at bio-interface. Biosens. Bioelectron..

[9] Mraović M., Muck T., Pivar M., Trontelj J., Pleteršek A. (2014). Humidity sensors printed on recycled paper and cardboard. Sensors.

[10] Romero F.J., Rivadeneyra A., Salinas-Castillo A., Ohata A., Morales D.P., Becherer M., Rodriguez N. (2019). Design, fabrication and characterization of capacitive humidity sensors based on emerging flexible technologies. Sens. Actuators B Chem..

[11] Boudaden J., Steinmaßl M., Endres H.-E., Drost A., Eisele I., Kutter C., Müller-Buschbaum P. (2018). Polyimide-based capacitive humidity sensor. Sensors.

[12] Schubert P.J., Navin J.H. (1985). A polyimide-based capacitive humidity sensor. IEEE Trans. Electron Devices.

[13] Matsuguchi M., Sadaoka Y., Sakai Y. (1991). A capacitive-type humidity sensor using cross-linked poly(methyl methacrylate) thin films. J. Electrochem. Soc..

[14] Ducéré V., Bernès A., Lacabanne C. (2005). A capacitive humidity sensor using cross-linked cellulose acetate butyrate. Sens. Actuators B Chem..

[15] Sadaoka Y., Comini E., Faglia G., Sberveglieri G. (2009). Chapter 3: Capacitive-type relative humidity sensor with hydrophobic polymer films. Solid State Gas Sensing.

[16] Zhou R., Li J., Jiang H., Li H., Wang Y., Briand D., Camara M., Zhou G., de Rooij N.F. (2019). Highly transparent humidity sensor with thin cellulose acetate butyrate and hydrophobic AF1600X vapor permeating layers fabricated by screen printing. Sens. Actuators B Chem..

[17] Fan Z., Zhi C., Wu L., Zhang P., Feng C., Deng L., Yu B., Qian L. (2019). UV/ozone-assisted rapid formation of high-quality tribological self-assembled monolayer. Coatings.

[18] Lin T.-Y., Pfeiffer T.T., Lillehoj P.B. (2017). Stability of UV/ozone-treated thermoplastics under different storage conditions for microfluidic analytical devices. RCS Adv..

[19] Lee J.-W., Hwang W. (2017). Simple fabrication of superoleophobic titanium surfaces via hierarchical microhorn/nanoporous structure growth by chemical acid etching and anodization. J. Alloys Compd..

[20] Kang Y., Ju S. (2018). Graphene-filter-mounted tin-oxide-nanowire-transistor for chemical sensor. Semicond. Sci. Technol..

[21] Lee Y., Cha S.H., Kim Y.-W., Choi D., Sun J.-Y. (2018). Transparent and attachable ionic communicators based on self-cleanable triboelectric nanogenerators. Nat. Commun..

[22] Li X.B., Larson S.D., Zyuzin A.S., Mamishev A.V. (2006). Design principles for multichannel fringing electric field sensors. IEEE Sens. J..

[23] Goswami M.P., Montazer B., Sarma U. (2019). Design and characterization of a fringing field capacitive soil moisture sensor. IEEE Trans. Instrum. Meas..

[24] Liu Y., Su C., Ren X., Fan C., Zhou W., Wang F., Ding W. (2014). Experimental study on surface modification of PET films under bipolar nanosecond-pulse dielectric barrier discharge in atmospheric air. Appl. Surf. Sci..

[25] Küchler F., Färber R., Franck C.M. Humidity and temperature effects on the dielectric properties of PET film. Proceedings of the 38th IEEE Electrical Insulation Conference (EIC).

[26] Angulo-Sherman A., Mercado-Uribe H. (2011). Dielectric spectroscopy of water at low frequencies: The existence of an isopermitive point. Chem. Phys. Lett..

[27] Choi J.M., Kim T.W. (2013). Humidity sensor using an air capacitor. Trans. Electr. Electron. Mater..

[28] Cular S. The Measurement and Uncertainty of Air Dielectric Capacitors from 1 kHz to 10 MHz. https://www.osti.gov/servlets/purl/1504063.

[29] Jensen K.R., Fojan P., Jensen R.L., Gurevich L. (2014). Water condensation: A multiscale phenomenon. J. Nanosci. Nanotechnol..

[30] Yang Q., Sun P.Z., Fumagalli L., Stebunov Y.V., Haigh S.J., Zhou Z.W., Grigorieva I.V., Wang F.C., Geim A.K. (2020). Capillary condensation under atomic-scale confinement. Nature.

[31] Yamada Y., Ikuta T., Nishiyama T., Takahashi K., Takata Y. (2014). Droplet nucleation on a well-defined hydrophilic–hydrophobic surface of 10 nm order resolution. Langmuir.

[32] Varanasi K.K., Hsu M., Bhate N., Yang W., Deng T. (2009). Spatial control in the heterogeneous nucleation of water. Appl. Phys. Lett..

[33] Kajiya T., Schellenberger F., Papadopoulos P., Vollmer D., Butt H.-J. (2016). 3D imaging of water-drop condensation on hydrophobic and hydrophilic lubricant-impregnated surfaces. Sci. Rep..

[34] Ranathunga D.T.S., Shamir A., Dai X., Nielsen S.O. (2020). Molecular dynamics simulations of water condensation on surfaces with tunable wettability. Langmuir.

